# 96 Decreasing Pain During Dressing Changes in a Trauma Burn Intensive Care Unit Through Virtual Reality

**DOI:** 10.1093/jbcr/iraf019.096

**Published:** 2025-04-01

**Authors:** Jamie Rossetto, Debra Eastman, Shannon Brunt, Lori Pelham

**Affiliations:** University of Michigan Trauma Burn Center; University of Michigan Health; Michigan Medicine; Michigan Medicine

## Abstract

**Introduction:**

Dressing changes, especially for burn patients, can be extremely painful. Literature confirms that the use of pain medications and virtual reality can alleviate pain during these procedures. Traditionally, patients have relied on music and pain medications, but advanced technology has not been widely implemented for distraction and pain management. This evidence-based project aims to reduce pain in adult patients undergoing wound care in the trauma burn center by utilizing virtual reality in comparison to medications and music.

**Methods:**

A comprehensive literature review was conducted, incorporating eighteen articles based on levels of evidence. The findings suggest that virtual reality reduces pain, decreases the need for pain medication during dressing changes, and enables nurses to clean wounds more effectively in less time. Baseline and post-implementation data were extracted, and staff received training on the use of virtual reality. Project outcomes were measured by the percentage of patients with pain scores greater than 7 one hour after wound care, the percentage requiring opioids within 30-60 minutes post-care, and the average amount of IV medications used during wound care, comparing pre- and post-virtual reality implementation.

**Results:**

The results indicated a 28% reduction in pain scores greater than 7/10, a 5% decrease in fentanyl use, and a 25% reduction in midazolam use. There was no change in opioid use 30-60 minutes post-care. Virtual reality effectively reduced pain and the need for intravenous medications during wound care.

**Conclusions:**

The use of virtual reality during dressing changes significantly reduces pain. Patients utilizing virtual reality report lower pain scores post-dressing change and often require fewer IV medications during the procedure.

**Applicability of Research to Practice:**

Implementing virtual reality during wound care enhances pain management for patients and reduces the need for sedation medications, thereby lowering the risk of complications from sedation and improving patient satisfaction. Future steps include collecting data on a larger scale and exploring the most effective virtual reality applications for patient care.

**Funding for the Study:**

N/A

